# Yeast Cells Exposed to Exogenous Palmitoleic Acid Either Adapt to Stress and Survive or Commit to Regulated Liponecrosis and Die

**DOI:** 10.1155/2018/3074769

**Published:** 2018-01-31

**Authors:** Karamat Mohammad, Paméla Dakik, Younes Medkour, Mélissa McAuley, Darya Mitrofanova, Vladimir I. Titorenko

**Affiliations:** Concordia University, Department of Biology, Montreal, QC, Canada H4B 1R6

## Abstract

A disturbed homeostasis of cellular lipids and the resulting lipotoxicity are considered to be key contributors to many human pathologies, including obesity, metabolic syndrome, type 2 diabetes, cardiovascular diseases, and cancer. The yeast *Saccharomyces cerevisiae* has been successfully used for uncovering molecular mechanisms through which impaired lipid metabolism causes lipotoxicity and elicits different forms of regulated cell death. Here, we discuss mechanisms of the “liponecrotic” mode of regulated cell death in *S. cerevisiae*. This mode of regulated cell death can be initiated in response to a brief treatment of yeast with exogenous palmitoleic acid. Such treatment prompts the incorporation of exogenously added palmitoleic acid into phospholipids and neutral lipids. This orchestrates a global remodeling of lipid metabolism and transfer in the endoplasmic reticulum, mitochondria, lipid droplets, and the plasma membrane. Certain features of such remodeling play essential roles either in committing yeast to liponecrosis or in executing this mode of regulated cell death. We also outline four processes through which yeast cells actively resist liponecrosis by adapting to the cellular stress imposed by palmitoleic acid and maintaining viability. These prosurvival cellular processes are confined in the endoplasmic reticulum, lipid droplets, peroxisomes, autophagosomes, vacuoles, and the cytosol.

## 1. Introduction

Some forms of cell death are classified as “programmed” cell death subroutines; they involve molecular machineries dedicated to commit cellular “suicide” that is aimed at providing certain benefits for development and/or survival of the entire organism [1–6]. Other forms of cell death are actively driven by molecular machineries that attempt to protect cells against certain stresses (without providing benefits for organismal development and/or survival); these forms are known as “regulated” cell death (RCD) subroutines [1, 3]. Cells commit RCD executed by a discrete molecular machinery because (1) the capacity of a molecular machinery dedicated to cell protection against a certain kind of stress is not sufficient to maintain cell viability if the intensity of such extracellular and/or intracellular stress exceeds a threshold and/or (2) molecular machineries driving some cellular processes that (directly or indirectly) contribute to cell protection against a certain kind of exogenous and/or endogenous stress are excessively activated, thereby generating products of these processes in concentrations that are lethal to the cell [1, 3, 7].

*S. cerevisiae* is a model organism most commonly and productively used for studying different forms of RCD elicited by perturbations in lipid metabolism [8–37]. The detailed knowledge of mechanisms underlying the molecular pathways of various modes of lipotoxic RCD in this yeast is therefore instrumental to our understanding of many human pathologies that are causally linked to dysregulated lipid metabolism, unbalanced lipid homeostasis, lipotoxicity, and lipid-induced cell death [31, 34, 37–41]. Among these human pathologies are obesity, metabolic syndrome, type 2 diabetes, insulin resistance, cardiovascular diseases, hepatic steatosis, liver cirrhosis, and cancer [34, 38–56].

The scope of this review is to analyze mechanisms underlying one of the modes of lipotoxic RCD. It has been discovered in the yeast *S. cerevisiae* and called “liponecrosis.” Liponecrotic RCD can be elicited by a short-term (for 2 h) treatment of yeast cells with exogenous palmitoleic acid (POA), a 16-carbon monounsaturated fatty acid (16 : 1 n-7) [57–59]. We describe different cellular processes that yeast cells exposed to POA use for stress adaptation and viability maintenance. We critically evaluate mechanisms (including POA-induced oxidative stress) through which yeast cells that are exposed to POA die of liponecrosis if the capacities of cellular processes for protection against POA-imposed stress become insufficient to maintain cell viability. We outline the most important unanswered questions and suggest directions for future research.

## 2. How Do Yeast Cells Die If Treated with POA and How Do They Mount a Protective Stress Response to Survive Such Treatment?

**Figure 1:**
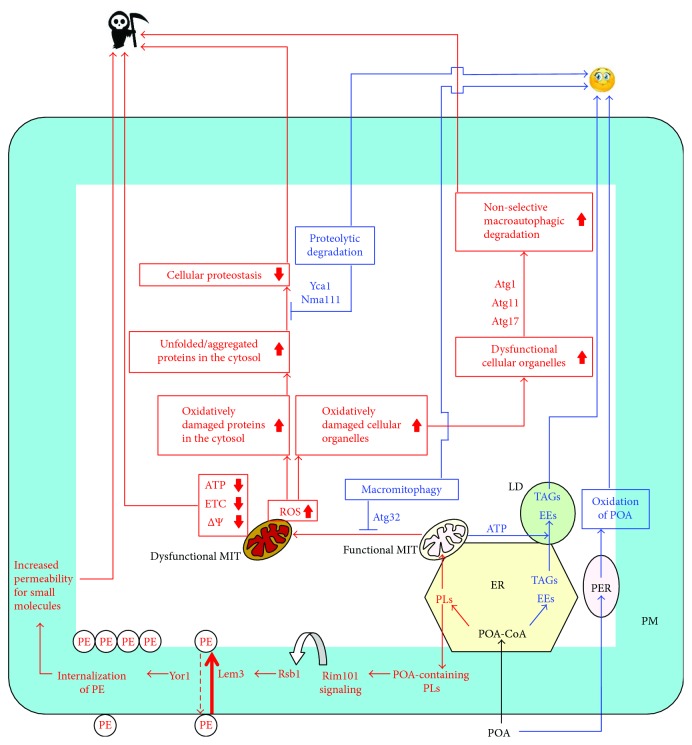
A model for how yeast cells exposed to exogenous palmitoleic acid (POA) either mount a protective stress response and survive or commit to POA-induced regulated liponecrosis and die. Yeast cells briefly exposed to POA can employ four different prosurvival processes to cope with the POA-induced cellular stress and maintain viability. These prosurvival cellular processes include the following: (1) an assimilation of POA into neutral lipids (triacylglycerols (TAGs) and ergosteryl esters (EEs)), in the endoplasmic reticulum (ER) and the subsequent deposition of these neutral lipids in lipid droplets (LD); (2) POA oxidation in peroxisomes (PER); (3) a macroautophagic degradation of dysfunctional or damaged mitochondria (MIT); and (4) a proteolytic degradation of oxidatively damaged, dysfunctional, unfolded, and aggregated proteins that accumulate in the cytosol of yeast cells. Arrows and names displayed in blue color denote prosurvival processes, metabolites, and proteins that protect yeast from POA-induced liponecrotic regulated cell death (RCD). Yeast cells briefly treated with POA can use four different pro-death processes to commit to POA-induced liponecrotic RCD and to execute this RCD subroutine. These pro-death cellular processes include the following: (1) a buildup of POA-containing phospholipids (PLs) in the PM and the ensuing increase in the permeability of the PM to small molecules; (2) the accumulation of POA-containing PLs in both mitochondrial membranes and the resulting decline in mitochondrial functionality, which is needed to support the prosurvival process of incorporating exogenous POA into neutral lipids; (3) a ROS-inflicted oxidative damage to mitochondria and other cellular organelles, which stimulates a nonselective macroautophagic degradation of many kinds of organelles; and (4) a ROS-imposed oxidative damage to cytosolic proteins, which impairs cellular proteostasis because it promotes an accumulation of oxidatively damaged, dysfunctional, unfolded, and aggregated proteins in the cytosol. Arrows and names displayed in red color denote pro-death processes, metabolites, and proteins that commit yeast to POA-induced RCD or execute this RCD subroutine. The up or down arrows in red color denote processes or metabolites whose intensities or concentrations are increased or decreased (resp.) in yeast cells briefly exposed to exogenous POA. See text for more details. ETC, mitochondrial electron transport chain; PE, phosphatidylethanolamine; PLs, phospholipids; PM, plasma membrane; ROS, reactive oxygen species; Δ*Ψ*, electrochemical potential across the inner mitochondrial membrane.

A model for the mechanism of liponecrotic RCD elicited by a short-term treatment of yeast with POA and for the mechanism protecting yeast from such RCD is schematically depicted in Figure 1.

Yeast cells that are briefly exposed to exogenous POA use the lipid-synthesizing and lipid-transporting enzymatic machineries of the endoplasmic reticulum (ER), mitochondria, lipid droplets (LDs), and the plasma membrane (PM) to incorporate this fatty acid into copious amounts of two classes of lipids [34, 59]. One of these POA-containing classes are the so-called “neutral” (uncharged) lipids triacylglycerols (TAGs) and ergosteryl esters (EEs), both of which are first produced in the ER and then deposited in LDs (Figure 1) [37, 40, 60, 61]. The other class are POA-containing phospholipids (Figure 1); they include (1) phosphatidic acid (PA), phosphatidylserine (PS), phosphatidylcholine (PC), and phosphatidylinositol (PI), all of which are synthesized only in the ER and then transferred to mitochondria through mitochondria-ER junctions and to the PM through PM-ER junctions [62–70]; (2) phosphatidylethanolamine (PE), which is produced from ER-derived PS in the inner and outer mitochondrial membranes (IMM and OMM, resp.) and then transferred to the ER through mitochondria-ER junctions and from the ER to the PM through PM-ER junctions [62, 63, 65, 69, 71–75]; and (3) cardiolipin (CL), a signature mitochondrial phospholipid which is generated from ER-derived PA in a series of reactions confined to the IMM and OMM [71, 74, 76–78]. It needs to be emphasized that genetic interventions weakening the incorporation of exogenously added POA into POA-containing phospholipids within the ER have been shown to increase cell resistance to POA-induced liponecrotic RCD [34, 59]. Thus, such incorporation is a pro-death process essential for the commitment of yeast to liponecrotic RCD in response to treatment with exogenous POA.

After being synthesized in the ER, the bulk quantities of POA-containing phospholipids in yeast cells committed to liponecrotic RCD amass in the PM (Figure 1) [34, 59]. Such accumulation of POA-containing phospholipids in the PM activates the alkaline pH- and lipid asymmetry-responsive Rim101 signaling pathway, which orchestrates a series of endocytic internalization and traffic events ultimately promoting transcription of the nuclear *RSB1* gene [79–87]. A protein product of this gene, Rsb1, is known to regulate the bidirectional active transport of PE across the PM bilayer; specifically, Rsb1 stimulates the Lem3-driven transport of PE from the outer monolayer of the PM to its inner monolayer and also slows down the Yor1-driven transport of PE in the opposite direction [86, 88–91]. These effects of Rsb1 elicit a depletion of PE in the outer monolayer of the PM, thereby markedly rising the permeability of the PM to small molecules (Figure 1) [34, 59]. Such increase in the permeability of the PM to small molecules has been shown to play an essential role in committing yeast to POA-induced liponecrotic RCD (Figure 1) [34, 59].

The bulk quantities of POA-containing phospholipids initially synthesized in the ER of yeast cells that are committed to liponecrotic RCD accumulate not only in the PM but also in both membranes enclosing mitochondria (Figure 1) [34, 59]. This buildup of POA-containing phospholipids in the IMM and OMM markedly weakens mitochondrial respiratory capacity, uncouples mitochondria respiratory chain from ATP synthesis, and lowers the electrochemical potential across the IMM (Figure 1) [34, 59]. The resulting decline in mitochondrial functionality plays an essential role in committing yeast to POA-induced liponecrotic RCD, likely because these dysfunctional mitochondria cannot produce enough ATP to support the energy-demanding, prosurvival process of incorporating exogenous POA into neutral lipids (see text below for discussion of this prosurvival process) (Figure 1) [34, 59].

The buildup of POA-containing phospholipids in the IMM and OMM of yeast committed to liponecrotic RCD not only impairs mitochondrial functionality but also considerably increases the intracellular concentration of reactive oxygen species (ROS) that are produced in mitochondria as by-products of respiration (Figure 1) [34, 59]. This rise of ROS concentrations elicits an oxidative damage to different types of molecules in two cellular locations, namely, to (1) protein and lipid components of mitochondria and other cellular organelles and (2) proteins in the cytosol, thereby causing their unfolding and aggregation (Figure 1) [34, 59]. Both these types of cellular oxidative damage are essential contributing factors either to the commitment of yeast to POA-induced liponecrotic RCD or to an execution of this RCD subroutine. Specifically, a massive breakdown of numerous oxidatively damaged and dysfunctional organelles through a nonselective macroautophagic degradation (which is choreographed by the phagophore assembly-specific serine/threonine protein kinase Atg1, adapter protein Atg11, and scaffold protein Atg17 [58, 59, 92–94]) plays a crucial role in executing POA-induced liponecrotic RCD (Figure 1) [34, 58, 59]. Moreover, the buildup of oxidatively damaged, dysfunctional, unfolded, and aggregated proteins in the cytosol of yeast cells treated with POA impairs cellular proteostasis, thus committing these cells to POA-induced liponecrotic RCD (Figure 1) [34, 59].

If the stress imposed by an exposure to POA does not exceed a toxic threshold, yeast cells can use at least four different processes to cope with this stress and maintain viability (Figure 1).

One of these prosurvival cellular processes is an assimilation of POA into neutral lipids (TAGs and EEs), which occurs in the ER and is followed by a buildup of POA-containing neutral lipids in LDs (Figure 1) [34, 58, 59]. This process lowers the extreme cellular stress caused by the accumulation of POA-containing phospholipids in the PM, IMM, and OMM because it attenuates the flow of POA into the pathways for the synthesis of POA-containing phospholipids [34, 58, 59]. The assimilation of POA into neutral lipids is essential for protecting yeast from POA-induced liponecrotic RCD, as demonstrated by the finding that genetic interventions weakening the incorporation of exogenously added POA into POA-containing neutral lipids increase the susceptibility of yeast to this subroutine of RCD [58].

Another prosurvival cellular process is the *β*-oxidation of POA in peroxisomes of yeast exposed to this monounsaturated free fatty acid (Figure 1) [34, 57–59]. Peroxisomal oxidation of POA mitigates POA-induced liponecrotic RCD because it weakens the incorporation of POA into phospholipids, thereby relieving the excessive cellular stress instigated by the buildup of POA-containing phospholipids in the PM, IMM, and OMM [34, 58, 59]. In support of an essential role of peroxisomal oxidation of POA in the protection of yeast from POA-induced liponecrotic RCD, yeast strains that carry the single-gene-deletion mutations *pex5Δ* and *fox1Δ* attenuating oxidative degradation of POA in peroxisomes are more susceptible to this mode of RCD than an otherwise isogenic wild-type strain [34, 57–59].

Macromitophagy, a macroautophagic degradation of dysfunctional or damaged mitochondria, is also a prosurvival process that allows yeast to cope with the POA-induced cellular stress [34, 58, 59]. Macromitophagy protects yeast from POA-induced liponecrotic RCD because, by selectively degrading dysfunctional mitochondria, it helps to maintain a population of functionally active mitochondria that are needed to generate enough ATP to support the prosurvival process of assimilating POA into neutral lipids (Figure 1) [34, 58, 59]. The *Atg32Δ*-dependent mutational block of macromitophagy impairs the accumulation of LD-deposited neutral lipids and sensitizes yeast to POA-induced liponecrotic RCD [58]; thus, macromitophagy plays an essential role in protecting yeast from this subroutine of RCD.

The degradation of oxidatively damaged, dysfunctional, unfolded, and aggregated proteins that accumulate in the cytosol of yeast cells treated with POA is another prosurvival process in these cells; this proteolytic degradation is catalyzed by the metacaspase Yca1 and serine protease Nma111, two protein components of the caspase-dependent apoptotic RCD pathway (Figure 1) [34, 59, 95–97]. This Yca1- and Nma111-driven proteolysis of oxidatively damaged, dysfunctional, unfolded, and aggregated proteins slows down a progression of POA-induced liponecrotic RCD because it allows to sustain efficient cellular proteostasis, thereby weakening proteostatic cellular stress (Figure 1) [34, 59]. In support of an essential role of such proteolysis in the protection of yeast from POA-induced liponecrotic RCD, lack of Yca1 or Nma111 increases the susceptibility of yeast to this mode of RCD [34, 59].

## 3. What Are the Relations among Different Processes Involved in Cell Death or Cell Adaptation in Yeast Treated with POA and How Is a Balance between Pro-death and Prosurvival Processes Regulated?

As outlined in the previous section, pro-death cellular processes in yeast treated with POA are direct or indirect due to the initial incorporation of this fatty acid into bulk quantities of POA-containing phospholipids. Two direct pro-death processes include the following: (1) the buildup of POA-containing phospholipids in the PM and the ensuing increase in the permeability of the PM to small molecules and (2) the accumulation of POA-containing phospholipids in both mitochondrial membranes and the resulting decline in mitochondrial functionality, which is needed to support the prosurvival process of incorporating exogenous POA into neutral lipids (Figure 1). Two other pro-death processes only indirectly caused the buildup of POA-containing phospholipids in both mitochondrial membranes because such buildup elicits a rise in the intracellular concentration of ROS initially produced in mitochondria. These indirect pro-death processes are as follows: (1) the ROS-inflicted oxidative damage to mitochondria and other cellular organelles, which stimulates a nonselective macroautophagic degradation of many kinds of organelles and (2) the ROS-imposed oxidative damage to cytosolic proteins, which impairs cellular proteostasis because it promotes the accumulation of oxidatively damaged, dysfunctional, unfolded, and aggregated proteins in the cytosol (Figure 1). Thus, the four pro-death processes relate because they all are initiated in response to the buildup of POA-containing phospholipids. We hypothesize that (1) the direct pro-death processes may precede in time the indirect ones and (2) the relative contribution of each direct or indirect pro-death process into POA-induced liponecrotic RCD may be defined by the relative rates with which POA-containing phospholipids are transferred from the ER to the PM and mitochondria, mitochondria generate ROS, mitochondria and other cellular organelles undergo ROS-inflicted oxidative damage, oxidatively damaged cellular organelles are subjected to nonselective macroautophagic degradation, cytosolic proteins are oxidatively damaged by mitochondrially produced ROS, and oxidatively damaged cytosolic proteins unfold and aggregate. In our hypothesis, none of the pro-death processes may be considered as an individual pro-death pathway. In contrast, our hypothesis posits that all four pro-death processes are likely to be nodes of a branched subnetwork that integrates the flow of POA-containing phospholipids from the ER to the PM and mitochondria, mitochondrial ROS formation, the ROS-imposed oxidative damage to organelles and cytosolic proteins, the nonselective macroautophagic breakdown of different kinds of oxidatively damaged organelles, and the unfolding and aggregation of oxidatively damaged proteins in the cytosol.

Our hypothesis further suggests that prosurvival processes are likely to be nodes of the same branched subnetwork integrating the four pro-death processes. Two direct prosurvival processes relieve the extreme cellular stress by preventing the buildup of POA-containing phospholipids in the PM and mitochondria; they include the following: (1) the assimilation of POA into neutral lipids in the ER and the subsequent buildup of POA-containing neutral lipids in LDs and (2) peroxisomal oxidation of POA (Figure 1). Two indirect prosurvival processes are activated to lower the extreme cellular stress created by the buildup of POA-containing phospholipids in both mitochondrial membranes and by the resulting decline in mitochondrial functionality and rise in mitochondrially produced ROS; they are as follows: (1) the selective macroautophagic degradation of oxidatively damaged and dysfunctional mitochondria, which helps to maintain a population of functionally active mitochondria generating sufficient quantities of ATP and producing ROS in nontoxic concentrations and (2) the Yca1- and Nma111-driven proteolysis of oxidatively damaged and aggregated cytosolic proteins, which allows to sustain efficient cellular proteostasis (Figure 1). Akin to pro-death processes, the four prosurvival processes relate because they all are stimulated in attempt to relieve the extreme cellular stress that is generated (directly or indirectly) by the initial incorporation of POA into POA-containing phospholipids. Our hypothesis posits that (1) the direct prosurvival processes may occur earlier than the indirect ones and (2) the relative contribution of each direct or indirect prosurvival process into cell protection from POA-induced liponecrosis may depend on the relative rates with which POA is assimilated into neutral lipids in the ER, POA-containing neutral lipids are transferred from the ER to LDs, POA is oxidized in peroxisomes, oxidatively damaged and dysfunctional mitochondria are subjected to selective macroautophagic degradation, and oxidatively damaged and aggregated cytosolic proteins undergo proteolytic degradation.

In sum, the above hypothesis posits the following: (1) the balance between different pro-death and prosurvival processes may be regulated by their relative rates and (2) these relative rates may be defined by the extracellular and/or intracellular concentrations of POA, nutrient availability, the metabolic state of a yeast cell, and the chronological age of a yeast cell.

## 4. Is the Subnetwork of Liponecrotic RCD Integrated into a Signaling Network Orchestrating Different RCD Scenarios in Yeast Cells?

Yeast cells undergoing POA-induced liponecrotic RCD exhibit characteristic morphological and biochemical traits [34, 58, 59]. Some of these traits are unique to liponecrotic RCD, whereas other traits are shared by this and certain other modes of RCD (Table 1).

While yeast cells committed to POA-induced liponecrotic RCD do not display such characteristic traits of apoptotic RCD as nuclear fragmentation and PS externalization within the PM bilayer, the metacaspase Yca1 and serine protease Nma111 play essential roles in both liponecrotic and caspase-dependent apoptotic modes of RCD [34, 59]. However, the roles Yca1 and Nma111 play in each of these two RCD modes are quite different (Table 1). As mentioned above, the Yca1- and Nma111-dependent proteolysis of oxidatively damaged, dysfunctional, unfolded, and aggregated proteins in the cytosol of yeast cells is a prosurvival process in POA-induced liponecrotic RCD [34, 59]. Such prosurvival role of Yca1 in sustaining efficient cellular proteostasis is well known [98–105]. In contrast, the Yca1- and Nma111-driven degradation of various cellular proteins is an executing, pro-death process in several caspase-dependent modalities of apoptotic RCD in yeast exposed to certain exogenous stimuli [95–97, 106–110].

While yeast cells undergoing POA-induced liponecrotic RCD do not display such hallmark trait of autophagic RCD as extreme cytoplasmic vacuolization instigated by a buildup of double-membrane vesicles called autophagosomes [34, 58, 59], both liponecrotic and autophagic modes of RCD (1) exhibit a nonselective massive degradation of various cellular organelles and (2) depend on the phagophore assembly-specific serine/threonine protein kinase Atg1 for executing these RCD modes (Table 1) [1, 58, 59, 111–113].

While yeast cells undergoing POA-induced liponecrotic RCD do not exhibit such hallmark feature of necrotic RCD as a severe fracture of the PM [34, 58, 59], both liponecrotic and necrotic modes of RCD display substantially increased permeability of the PM to small molecules (Table 1) [31, 58, 59, 114–116].

A trait which is unique to POA-induced liponecrotic RCD is a buildup of POA-containing neutral lipids in numerous LDs, a feature that has not been reported for apoptotic, autophagic, or necrotic subroutine of RCD (Table 1) [1, 58, 59, 96, 111–113, 115].

Because POA-induced liponecrotic RCD has several different traits in common with apoptotic, autophagic, and necrotic modes of RCD, we hypothesize that the molecular subnetwork of POA-induced liponecrotic RCD is integrated into a signaling network that orchestrates different RCD scenarios in yeast cells. Other pathways and subnetworks integrated into this signaling network may include apoptotic, autophagic, and necrotic pathways and subnetworks of RCD. In our hypothesis, the molecular subnetwork of POA-induced liponecrotic RCD only partially overlaps with apoptotic, autophagic, and necrotic RCD pathways and subnetworks of the network. Our hypothesis satisfactorily explains the observed existence of several proteins that are common to liponecrotic, apoptotic, autophagic, and necrotic modes of RCD [34, 58, 59]. Furthermore, as our hypothesis suggests, some of the morphological and biochemical traits characteristic of POA-induced liponecrotic RCD are shared by this mode of RCD and other (i.e., apoptotic, autophagic, and necrotic) RCD modes integrated into the network [34, 58, 59]. Moreover, in agreement with our hypothesis on only a partial overlap between liponecrotic and other pathways and subnetworks of RCD, at least one trait characteristic of liponecrotic RCD is unique to this mode of RCD; this trait is the accumulation of POA-containing neutral lipids in many LDs [34, 58, 59].

Our hypothesis on the existence of an RCD signaling network orchestrating different RCD scenarios in yeast cells is reminiscent of the hypothesis on the global programmed cell death (PCD) network that has been proposed and then confirmed for mammalian cells [117–120]. A systems biology platform has been developed for defining the topology of such network operating in mammalian cells; this platform employs cell biological and computational approaches for measuring and computing the effects of single and double genetic interventions on the molecular events characteristic of different PCD modes that are integrated into the network [119]. The use of such platform, possibly in combination with powerful tools of proteomic and metabolomic analyses recently applied for molecular analyses of RCD in yeast [104, 105], will allow to test our hypothesis on the global RCD signaling network in yeast and, perhaps, to dissect the architecture of such network in the near future.

## 5. Does Liponecrotic RCD Contribute to Yeast Chronological Aging?

POA-induced liponecrotic RCD is an age-related mode of RCD, as the susceptibility of a population of yeast cells to POA-induced liponecrosis increases with the chronological age of this population [34, 58, 59, 121]. Furthermore, the susceptibility of yeast cells to POA-induced liponecrotic RCD can be significantly decreased by some aging-delaying dietary and pharmacological interventions. These interventions include caloric restriction (CR) and lithocholic bile acid (LCA), each implemented at the time of cell inoculation into growth medium [57, 60, 121].

Our recent unpublished findings indicate that in yeast cultured under non-CR conditions on 1% or 2% glucose, the risk of age-related death depends not only on the POA-induced liponecrotic mode of RCD but also on ROS-induced apoptotic RCD mode. Moreover, we found that the liponecrotic and apoptotic modes of RCD have different relative contributions to age-related death of non-CR yeast at different periods of chronological lifespan (CLS). The apoptotic mode of RCD predominates during diauxic (D) phase, apoptotic and liponecrotic RCD modes equally increase the risk of death during post-diauxic (PD) phase, whereas the liponecrotic mode of RCD prevails during stationary (ST) phase of culturing under non-CR conditions (our unpublished data). The longevity-defining mode of liponecrotic RCD is elicited by the accumulation of POA and other free fatty acids in chronologically aging non-CR yeast cells that progress through PD and ST phases of culturing (our unpublished data). In contrast, the longevity-defining mode of apoptotic RCD is caused by the rapid decline of mitochondrial functionality and rise of mitochondrially generated ROS in chronologically aging non-CR yeast cells progressing through D and PD phases of culturing (our unpublished data). CR diet, which is implemented by culturing yeast on 0.2% or 0.5% glucose, decreases the risk of age-related death by attenuating liponecrotic and apoptotic RCD modes during D, PD, and ST phases; these effects of CR are due to its abilities to (1) decrease free fatty acid (including POA) concentrations during PD and ST phases of culturing and to (2) improve mitochondrial functionality and to lessen concentrations of mitochondrially generated ROS during D and PD phases of culturing (our unpublished data).

LCA is a geroprotective chemical compound that delays yeast chronological aging mainly under CR conditions [57]. LCA exhibits the following effects on yeast susceptibility to POA-induced liponecrotic RCD: (1) it decreases such susceptibility only if added to growth medium at the time of cell inoculation, during logarithmic (L) or D phase of culturing; (2) it increases such susceptibility if added during PD phase; and (3) it has no effect on such susceptibility if added during ST phase [121]. Taken together, these findings suggest that liponecrotic RCD may be an essential longevity-limiting (i.e., proaging) factor in chronologically “young” yeast, may somehow contribute to longevity extension (i.e., aging delay) in chronologically “middle-aged” yeast, and may have no influence on longevity (i.e., on the pace of aging) of chronologically “old” yeast. Noteworthy, all these age-related variations in yeast susceptibility to POA-induced liponecrotic RCD coincide with age-related changes in yeast resistance to chronic oxidative, thermal, and osmotic stresses [121]. In the future, it would be important to explore mechanisms that underlie the observed age-related coincidence between yeast susceptibility to POA-induced liponecrotic RCD and yeast resistance to long-term stresses. Moreover, it remains to be determined if and how the concentrations of endogenously produced free fatty acids (including POA) influence the extent of liponecrotic RCD at different stages of yeast chronological aging.

Of note, LCA decreases yeast susceptibility to the mitochondria-controlled, ROS-induced mode of apoptotic RCD if added to growth medium at the time of cell inoculation and during L, D, PD, or ST phase of culturing [121]. In yeast cultured under CR conditions, exogenous LCA enters cells, is sorted to mitochondria, amasses primarily in the IMM and also resides in the OMM, alters the concentrations of certain mitochondrial membrane phospholipids, elicits a major enlargement of mitochondria, significantly decreases mitochondrial number, prompts an intramitochondrial accumulation of cristae disconnected from the IMM, triggers substantial alterations in mitochondrial proteome, decreases the frequencies of deletion and point mutations in mitochondrial DNA, and leads to changes in vital aspects of mitochondrial functionality [66, 68, 122, 123]. In the future, it would be important to explore how all these aging-delaying effects of LCA are linked to yeast susceptibility to the mitochondria-controlled, ROS-induced mode of apoptotic RCD at different stages of chronological aging.

In sum, it is conceivable that liponecrotic and apoptotic modes of RCD may have different effects on yeast CLS at different periods of life. This is similar to the “P” (“big P”) and “p” (“small p”) modes of death in the nematode *Caenorhabditis elegans*, which define lifespan earlier or later in life (resp.) [124]. The P mode of death is manifested as a substantial enlargement of the posterior pharyngeal bulb caused by intensified pharyngeal pumping, whereas the p mode of death is due to the complete atrophy of pharynx [124].

## 6. How Does Liponecrotic RCD Differ from Other Modes of Lipotoxic RCD in Yeast?

Several exogenously added lipids [8–16], as well as different genetic [11–13, 15–25] and pharmacological [24, 26–30] interventions that impair certain aspects of lipid metabolism, have been shown to elicit apoptotic and/or necrotic modes of lipotoxic RCD in yeast. These modes have been extensively reviewed elsewhere [31, 32, 37, 41, 125]. In brief, yeast cells committed to POA-induced liponecrotic RCD exhibit a unique combination of morphological and biochemical traits that is not characteristic of any of these other modes of lipotoxic RCD. Moreover, some of these other modes of lipotoxic RCD differ from each other with respect to (1) structural and/or functional features of yeast committed to a particular mode of RCD; (2) classes of lipids whose concentrations are altered (or are expected to be altered) in yeast committed to a particular mode of RCD; and (3) proteins that are involved in committing to and/or executing a particular mode of RCD [8–32, 37, 41, 125].

Altogether, these findings further support our hypothesis (which is outlined in Section 4) on the possible existence of a global signaling network that integrates partially overlapping molecular pathways and subnetworks of lipotoxic RCD, each pathway and subnetwork being differently responsive to certain perturbations in diverse aspects of lipid metabolism within a yeast cell. The key challenge for the future is to explore mechanisms through which such perturbations in lipid metabolism (1) modulate individual molecular pathways and subnetworks of lipotoxic RCD and (2) orchestrate the integration of these individual pathways and subnetworks into the global signaling network of lipotoxic RCD. To address this challenge, the systems biology platform (which is discussed in Section 4) exploited for mammalian cells [119] can be used in combination with proteomic and metabolomic analyses of molecular signatures [104, 105] characteristic of different lipotoxic RCD modes.

## 7. Conclusions

To cope with the lipotoxic stress imposed by an exposure to POA, *S. cerevisiae* cells use several different mechanisms to mount a protective stress response and maintain viability. This complex stress response consists in remodeling of at least four cellular processes. If the POA-induced lipotoxic stress exceeds a threshold, yeast cells commit suicide that is assisted by a complex molecular machinery. This molecular machinery alters the spatiotemporal dynamics of several cellular processes to execute a liponecrotic subroutine of RCD. The liponecrotic mode of POA-induced RCD plays an essential role in defining longevity of chronologically aging yeast, likely in coordination with an apoptotic mode of RCD. The molecular subnetwork of POA-induced liponecrotic RCD may be integrated into a global signaling network of partially overlapping molecular pathways and subnetworks, each executing a different mode of lipotoxic or nonlipotoxic RCD.

## Figures and Tables

**Table 1:** Some of the morphological and biochemical traits characteristic of palmitoleic acid- (POA-) induced liponecrotic regulated cell death (RCD) are unique to this mode of RCD, whereas other traits are shared by this mode and other (i.e., caspase-dependent apoptotic, autophagic, and necrotic) RCD modes. LDs, lipid droplets; PM, plasma membrane; PS, phosphatidylserine.

Trait	Caspase-dependent apoptotic RCD [references]	Autophagic RCD [references]	Necrotic RCD [references]	POA-induced liponecrotic RCD [references]
Nuclear fragmentation	+ [126]	−	−	− [58]
PS externalization within the PM	+ [126]	−	−	− [58]
Role of Yca1 and Nma111	+ (pro-death role) [106, 107]	−	−	+ (prosurvival role) [59]
Excessive cytoplasmic vacuolization	−	+ [127]	−	− [58]
Massive degradation of various cellular organelles	−	+ [127]	−	+ [58]
Rupture of the PM	−	−	+ [114]	− [58]
Permeability of the PM to small molecules	−	−	+ [114]	+ [59]
Excessive accumulation of LDs	−	−	−	+ [58]
